# A network meta-analysis of therapeutic outcomes after new image technology-assisted transurethral resection for non-muscle invasive bladder cancer: 5-aminolaevulinic acid fluorescence vs hexylaminolevulinate fluorescence vs narrow band imaging

**DOI:** 10.1186/s12885-015-1571-8

**Published:** 2015-08-01

**Authors:** Joo Yong Lee, Kang Su Cho, Dong Hyuk Kang, Hae Do Jung, Jong Kyou Kwon, Cheol Kyu Oh, Won Sik Ham, Young Deuk Choi

**Affiliations:** 1 Department of Urology, Severance Hospital, Urological Science Institute, Yonsei University College of Medicine, Seoul, Korea; 2 Department of Urology, Gangnam Severance Hospital, Urological Science Institute, Yonsei University College of Medicine, Seoul, Korea; 3 Department of Urology, Yangpyeong Health Center, Yangpyeong, Korea; 4 Department of Urology, Haeundae Paik Hospital, Inje University College of Medicine, Busan, Korea; 5 Department of Urology, Clinical Trial Center for Medical Devices, Severance Hospital, Urological Science Institute, Yonsei University College of Medicine, 50-1 Yonsei-ro, Seodaemun-gu, Seoul, 120-752 Korea

**Keywords:** Urinary bladder neoplasms, Photochemotherapy, Narrow band imaging, Meta-analysis, Bayes theorem

## Abstract

**Background:**

This study included a network meta-analysis of evidence from randomized controlled trials (RCTs) to assess the therapeutic outcome of transurethral resection (TUR) in patients with non-muscle-invasive bladder cancer assisted by photodynamic diagnosis (PDD) employing 5-aminolaevulinic acid (5-ALA) or hexylaminolevulinate (HAL) or by narrow band imaging (NBI).

**Methods:**

Relevant RCTs were identified from electronic databases. The proceedings of relevant congresses were also searched. Fifteen articles based on RCTs were included in the analysis, and the comparisons were made by qualitative and quantitative syntheses using pairwise and network meta-analyses.

**Results:**

Seven of 15 RCTs were at moderate risk of bias for all quality criteria and two studies were classified as having a high risk of bias. The recurrence rate of cancers resected with 5-ALA-based PDD was lower than of those resected using HAL-based PDD (odds ratio (OR) = 0.48, 95 % confidence interval (CI) [0.26–0.95]) but was not significantly different than those resected with NBI (*OR* = 0.53, 95 % CI [0.26–1.09]). The recurrence rate of cancers resected using HAL-based PDD versus NBI did not significantly differ (*OR* = 1.11, 95 % CI [0.55–2.1]). All cancers resected using 5-ALA-based PDD, HAL-based PDD, or NBI recurred at a lower rate than those resected using white light cystoscopy (WLC). No difference in progression rate was observed between cancers resected by all methods investigated.

**Conclusions:**

The recurrence rate of some bladder cancers can be decreased by the implementation of either PDD- and NBI-assisted TUR; in real settings, clinicians should consider replacing WLC as the standard imaging technology to guide TUR.

## Background

The conventional therapy for non-muscle invasive bladder cancer (NMIBC) is based on transurethral resection (TUR) combined with post-operational chemotherapy or immunotherapy with Bacille Calmette-Guérin [1]. Conventional white light cystoscopy (WLC) has been the standard method for detecting urothelial carcinoma during TUR [2]. However, the sensitivity and specificity of WLC is not entirely satisfactory [3]. Flat malignant lesions including carcinoma in situ (CIS) are difficult to visualize and distinguish from benign inflammatory lesions.

New imaging technologies including photodynamic diagnosis (PDD) and narrow band imaging (NBI) have recently been introduced; these technologies enhance bladder cancer visualization to improve diagnostic accuracy and the thoroughness of resection. PDD involves the instillation of photoactive porphyrin precursors such as 5-aminolaevulinic acid (5-ALA) or hexylaminolevulinate (HAL), which are metabolized to the photoactive compound intracellularly and then emit red fluorescence under blue light. NBI filters white light into two discrete bands in the blue and green spectrums that penetrate tissue only superficially but are strongly absorbed by hemoglobin. Both PDD and NBI are macroscopic modalities and can thus survey a large area of bladder mucosa in a manner similar to WLC, while providing additional contrast enhancement to highlight suspicious lesions and distinguish them from surrounding, noncancerous mucosa. Several studies have demonstrated that PDD and NBI are more sensitive than WLC in detecting small papillary bladder tumors and CIS, thus improving tumor detection rates and decreasing residual tumor rates. This study assessed the therapeutic outcome of PDD- or NBI-assisted TUR in patients with NMIBC via a network meta-analysis of evidence from randomized controlled trials (RCTs).

## Methods

### Inclusion criteria

Published RCTs that met the following criteria were included: (i) a study design that included measurement of the clinical efficacy of PDD or NBI and compared it with that of WLC in patients with suspected or confirmed NMIBC, (ii) a match between the baseline characteristics of patients from two groups, including the total number of subjects and the values of each index, (iii) the performance of the procedure under general anesthesia, spinal anesthesia, or combined spinal–epidural anesthesia, (iv) the assessment of at least one of the following outcomes: recurrence rate defined as the number of bladder cancer recurrences after initial TUR, progression rate defined as the number of patients with disease progression into muscle invasive bladder cancer during the follow-up period, or time until first recurrence, defined as the time until bladder cancer recurrence after initial TUR, and (v) accessibility to the study’s full text in English. When two or more studies reported on a group of patients at the same institution during an overlapping time period, only the study with the longest follow-up period was included. This report was prepared in compliance with Preferred Reporting Items for Systematic Reviews and Meta-Analyses (PRISMA) statement (accessible at http://www.prisma-statement.org/) [4].

### Search strategy

A literature search was performed across all publications prior to 31 December 2013 in PubMed, and EMBASE^™^ online databases. A cross-reference search of eligible articles was performed to identify additional studies not found by the computerized search. A combination of the following MeSH terms and keywords was used: fluorescence cystoscopy, photodynamic diagnoses, narrow, imaging, bladder cancer/tumor, white light cystoscopy, and randomized controlled trial.

### Data extraction

One researcher (J.Y.L.) screened the titles and abstracts identified by the search strategy. The other two researchers (D.H.K. and K.S.C.) independently assessed each paper’s full text to determine whether a paper met the inclusion criteria. The databases were designed to include the most relevant data with respect to author, year of publication, patient demographics, treatments, recurrence and progression outcomes, and inclusion of a reference standard. Disagreements were resolved by discussion until a consensus was reached or by arbitration employing another researcher (Y.D.C.).

### Study quality assessment and publication bias

Once the final group of articles was agreed upon, two researchers (J.Y.L. and D.H.K.) independently examined the quality of each article using the Cochrane’s risk of bias as a quality assessment tool for RCTs. The assessment includes assigning a judgment of “yes,” “no,” or “unclear” for each domain to designate a low, high, or unclear risk of bias, respectively. If one or no domain was deemed “unclear” or “no,” the study was classified as having a low risk of bias. If four or more domains are deemed “unclear” or “no,” the study was classified as having a high risk of bias. If two or three domains were deemed “unclear” or “no,” the study was classified as having a moderate risk of bias [5]. Publication bias was examined using funnel plots. In the absence of publication bias, this method assumes that the largest studies will be plotted near the average and that smaller studies will be spread evenly on both sides of the average, creating a roughly funnel-shaped distribution. Deviation from this shape can indicate publication bias. Quality assessment and investigation of publication bias were carried out using Review Manager 5 (RevMan 5.2.3, Cochrane Collaboration, Oxford, UK).

### Heterogeneity tests

Heterogeneity among the studies was explored using the Q-statistic and Higgins’ I^2^ statistic [6]. An I^2^ measures the percentage of total variation due to heterogeneity rather than chance across studies and is calculated as follows:

$$ {\mathrm{I}}^2=\frac{\mathrm{Q}\hbox{-} \mathrm{d}\mathrm{f}}{\mathrm{Q}}\times 100\% $$

where “Q” is Cochran’s heterogeneity statistic and “df” indicates the degrees of freedom.

An I^2^ ≥ 50 % was considered to represent substantial heterogeneity. For the Q statistic, heterogeneity was deemed to be significant for *p* less than 0.10 [7]. If there was evidence of heterogeneity, the data were analyzed using a random-effects model. Studies in which positive results were confirmed were assessed with a pooled specificity with 95 % CIs.

### Statistical analysis

Outcome variables measured at specific time points were compared in terms of odds ratios (OR) or mean differences with 95 % CIs using a network meta-analysis. Each analysis was based on non-informative priors for effect sizes and precision. Convergence and lack of auto-correlation were checked and confirmed after four chains and a 50,000-simulation burn-in phase; finally, direct probability statements were derived from an additional 100,000-simulation phase. Calculation of the probability that each stent has the lowest rate of clinical events was performed using Bayesian Markov Chain Monte Carlo modeling. Sensitivity analyses were performed by repeating the main computations using a fixed effect method. Model fit was appraised by computing and comparing estimates for deviance and information criterion. The existence of small study effects or publication bias was assessed by visual inspection of funnel plots for pairwise meta-analysis. All statistical analyses were performed using Review Manager 5 and R (R version 3.0.2, R Foundation for Statistical Computing, Vienna, Austria; http://www.r-project.org).

## Results

### Eligible studies

**Fig. 1:**
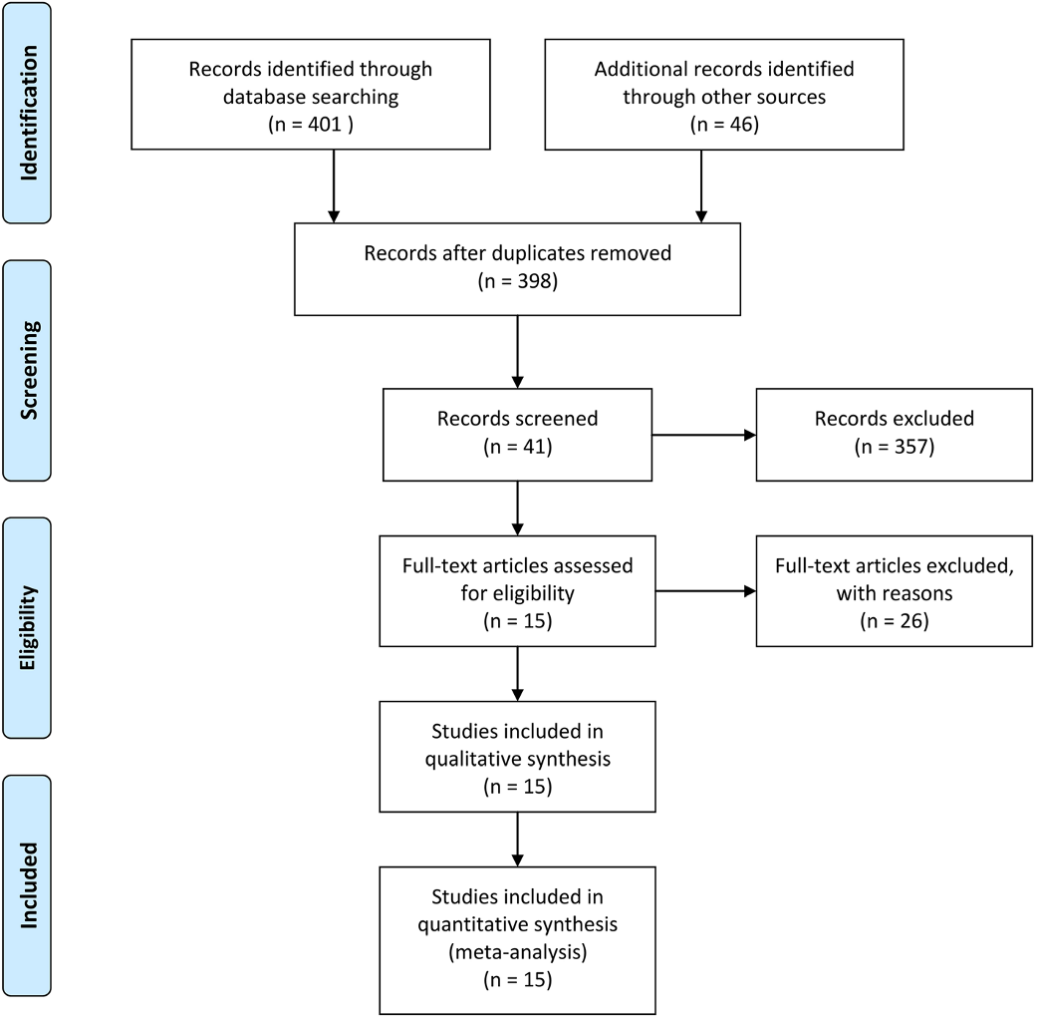
Flow diagram of evidence acquisition. Fifteen studies were ultimately included in the qualitative and quantitative synthesis using pairwise and network meta-analyses

The database search found 41 articles covering 398 studies for potential inclusion in the meta-analysis. Twenty-six articles were excluded according to the inclusion/exclusion criteria; 21 articles were retrospective models and 5 articles were reported as case series. The remaining 15 articles were included in the qualitative and quantitative synthesis using pairwise and network meta-analyses (Fig. 1).

**Fig. 2:**
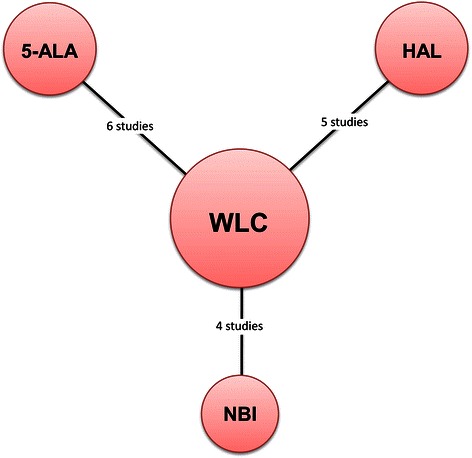
Network plots for included studies. Six studies compared TUR using 5-ALA-based PDD versus TUR with WLC. Five trials reported on therapeutic outcomes after TUR with HAL-based PDD versus TUR with WLC. Four studies included two arms of TUR with NBI and WLC were published

Data corresponding to confounding factors derived from each study are summarized in Table 1 [8–22]. These studies covered therapeutic outcomes of TUR assisted by three different types of PDD or NBI versus WLC (Fig. 2).

**Table 1:** Studies enrolled in this meta-analysis

Study	Year	Study design	Method	Cases		Follow-up (mos.)		Recurrence (%)		Progression (%)		Quality assessment^a^
				NIT	WLC	NIT	WLC	NIT	WLC	NIT	WLC	
Riedl [8]	2001	RCT	5-ALA	51	51	NA	NA	22 (45.8)	31 (66)	1 (2)	1 (2)	3
Filbeck [9]	2002	RCT	5-ALA	88	103	21	21	10 (11.4)	29 (28.2)	2 (2.3)	2 (1.9)	3
Kriegmair [10]	2002	RCT	5-ALA	65	64	NA	NA	17 (32.7)	26 (53.1)	NA	NA	2
Babjuk [11]	2005	RCT	5-ALA	60	62	21	22	5 (8.3)	23 (37.1)	5 (8.3)	5 (8.1)	3
Schumacher [12]	2010	RCT	5-ALA	138	141	12	12	NA	NA	14 (10.1)	15 (10.6)	1
Stenzl [13]	2011	RCT	5-ALA	271	280	12	21	128 (47.2)	157 (56.1)	5 (1.8)	7 (2.5)	0
Dragoescu [14]	2011	RCT	HAL	22	22	9	9	4 (18.2)	10 (45.5)	1 (4.5)	2 (9.1)	3
Hermann [15]	2011	RCT	HAL	77	68	12	12	18 (30.5)	35 (47.3)	14 (34.1)	17 (37.8)	3
Stenzl [16]	2010	RCT	HAL	183	176	12	12	NA	NA	19 (10.4)	19 (10.8)	1
Geavlete [17]	2012	RCT	HAL	125	114	24	24	39 (66.1)	52 (70.3)	5 (4)	8 (7)	1
Karaolides [18]	2012	RCT	HAL	41	45	18	18	7 (17.1)	18 (40)	NA	NA	4
Geavlete [19]	2012	RCT	NBI	110	110	12	12	7 (6.4)	16 (14.5)	NA	NA	3
Montanari [20]	2012	RCT	NBI	47	45	NA	NA	16 (34)	22 (48.9)	NA	NA	4
Naselli [21]	2012	RCT	NBI	76	72	12	12	25 (32.9)	37 (51.4)	NA	NA	0
Lee [22]	2014	RCT	NBI	33	35	16	15	5 (15.2)	8 (22.9)	1 (3)	2 (5.7)	1

*NIT* new image technology, *WLC* white light cystoscopy, *RCT* randomized controlled trial, *NA* not applicable, *5-ALA* 5-aminolaevulinic acid, *HAL* hexylaminolevulinate, *NBI* narrow band imaging

^a^Quality assessment was based on Cochrane’s risk of bias as a quality assessment tool for RCTs. If four or more domains are deemed “unclear” or “no,” the study was classified as having a high risk of bias. If two or three domains were deemed “unclear” or “no,” the study was classified as having a moderate risk of bias

### Quality assessment and publication bias

**Fig. 3:**
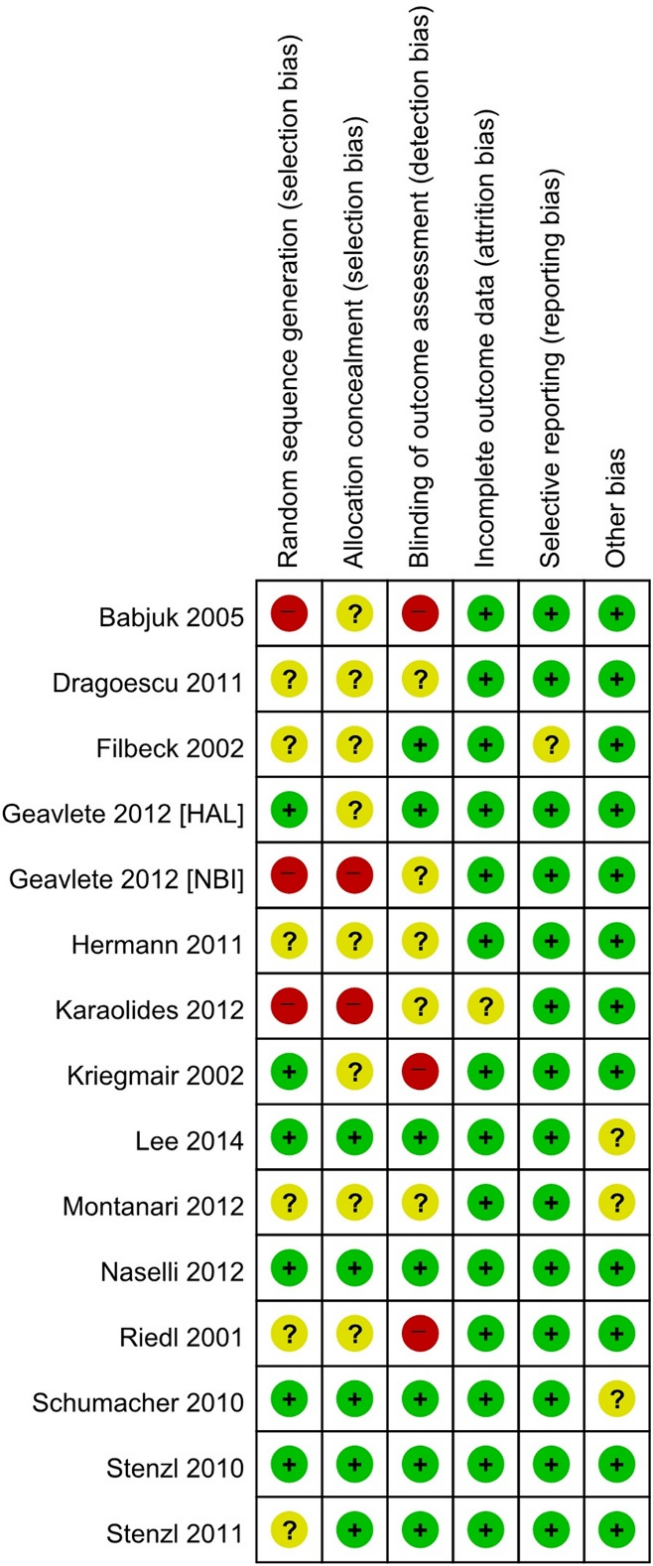
Risk of bias summary. Review authors’ judgments for each risk of bias item for each included study. Green; low risk of bias, Red; high risk of bias and Yellow; unclear of risk of bias

**Fig. 4:**
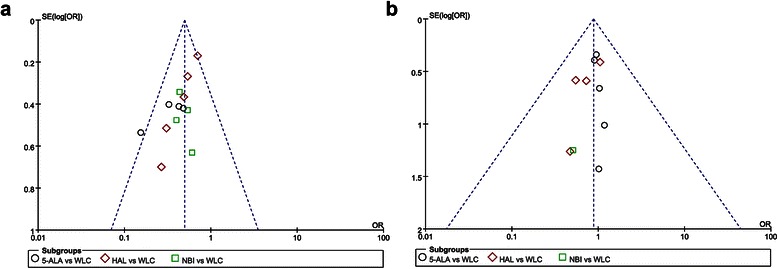
Funnel plots on recurrence (**a**) and progression rates (**b**). Little evidence of publication bias was demonstrated by visual or statistical examination of the funnel plots

Figure 3 presents the details of quality assessment, as measured by the Cochrane Collaboration risk-of-bias tool. Seven trials exhibited a moderate risk of bias for all quality criteria and two studies were classified as having a high risk of bias (Table 1). The most common risk factor for quality assessment was risk or insufficient information concerning allocation concealment and the second most common concerned random sequence generation. Most recently published studies exhibited low risk for quality assessment. In terms of cancer recurrence and progression rate, little evidence of publication bias was observed on visual or statistical examination of the funnel plots (Fig. 4).

### Heterogeneity assessment

**Fig. 5:**
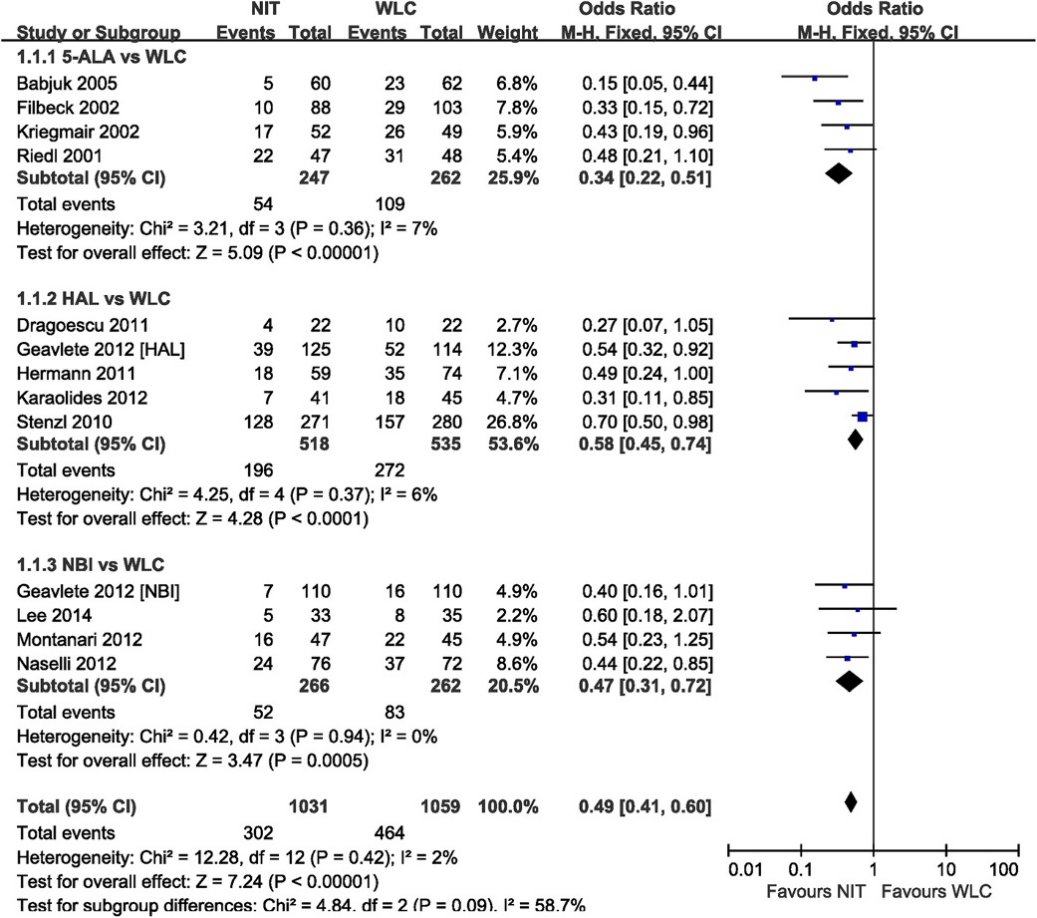
Pairwise meta-analysis for recurrence rate. 5-ALA- and HAL-based PDD, and NBI-guided TUR demonstrated lower recurrence rate than WLC

**Fig. 6:**
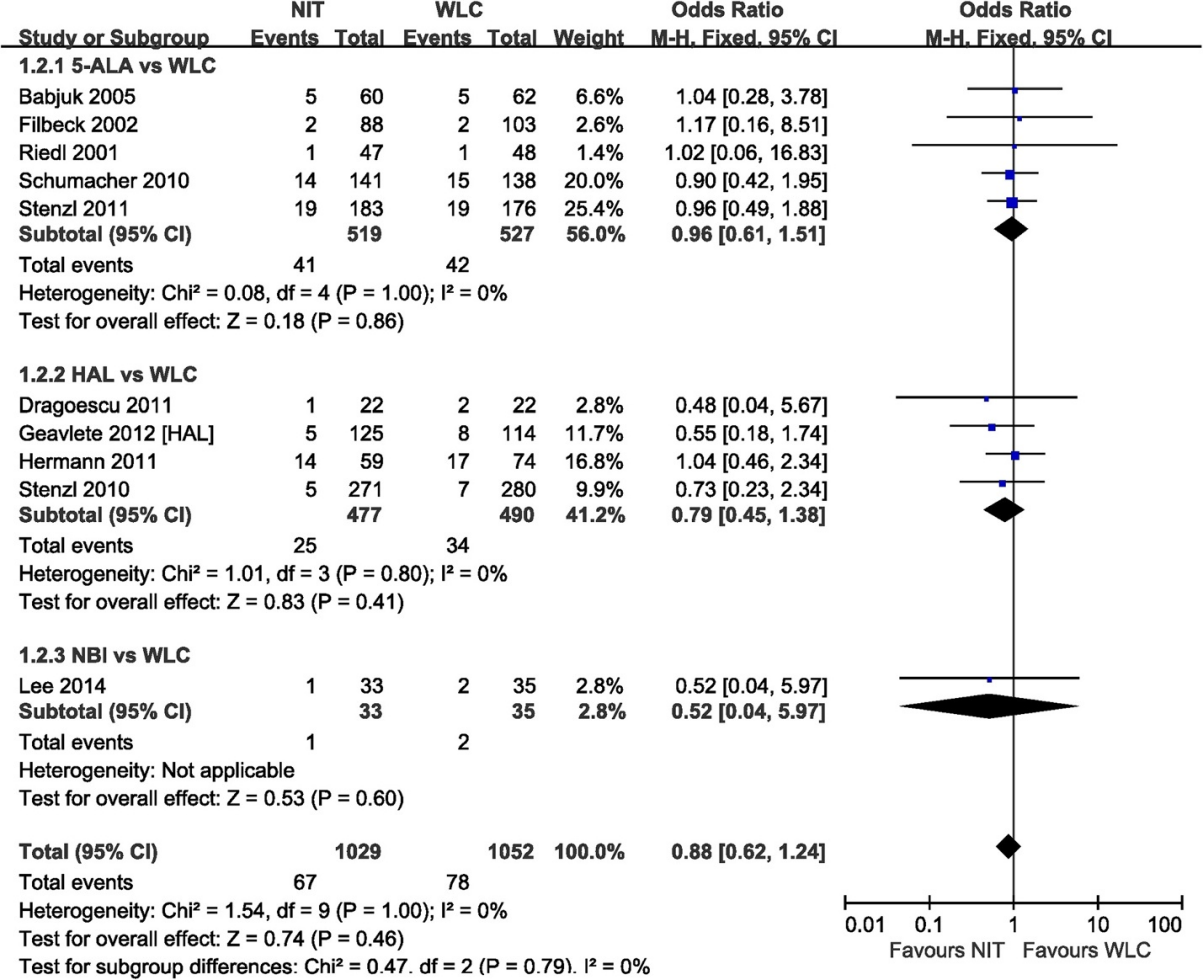
Pairwise meta-analysis for progression rate. No difference in progression rate was observed between cancers resected by all methods investigated

Forest plots of pairwise meta-analyses are shown in Figs. 5 and 6. A heterogeneity test for recurrence rate showed the following: *χ*^2^ = 3.21 with 3 df (*P* = 0.36) and *I*^*2*^ = 7 % in the analysis of TUR assisted by 5-ALA-based PDD versus WLC; *χ*^2^ = 4.25 with 4 df (*P* = 0.37) and *I*^*2*^ = 6 % in the analysis of TUR assisted by HAL-based PDD versus WLC; and *χ*^2^ = 0.42 with 3 df (*P* = 0.94) and I^2^ = 2 % in the meta-analysis of diagnosis by NBI versus WLC. In the analysis of progression rate, a heterogeneity test also demonstrated homogeneity with *χ*^2^ = 0.08 with 4 df (*P* = 1.00) and *I*^*2*^ = 0 % in TUR assisted by 5-ALA-based PDD versus WLC and *χ*^2^ = 1.01 with 3 df (*P* = 0.80) and *I*^*2*^ = 0 % in TUR assisted by HAL-based PDD versus WLC. Because there were no heterogeneities in these forest plots, the fixed effect models were applied using the Mantel–Haenszel method (Figs. 5 and 6).

### Pairwise meta-analysis of rates of recurrence and progression

The forest plot using the fixed effect model showed an OR of 0.34 (95 % CI [0.22–0.51], *P* < 0.001) between the recurrence rate of cancers resected using 5-ALA-based PDD versus WLC. Pairwise meta-analysis of cancers resected using HAL-based PDD versus WLC resulted in an OR of 0.58 (95 % CI [0.45–0.74], *P* < 0.001). According to the forest plot for recurrence rate, NBI-guided TUR was also superior to that using WLC, with an OR of 0.47 (95 % CI [0.31–0.72], *P* < 0.001) (Fig. 5). However, in terms of progression rate, cancers resected using 5-ALA-based PDD, HAL-based PDD, or NBI did not significantly differ from those cancers resected using WLC (all *P* > 0.05) (Fig. 6).

### Network meta-analysis for rates of recurrence and progression

**Fig. 7:**
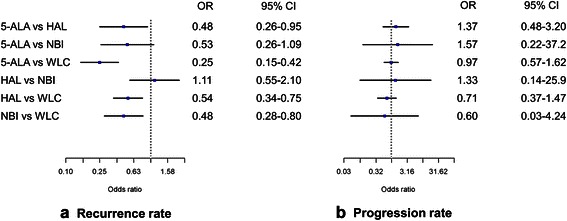
Network meta-analysis for recurrence and progression rates. **a** The recurrence rate of cancers resected using 5-ALA-based PDD was lower than that of those cancers resected using HAL-based PDD and was not significantly different from those resected using NBI. The use of 5-ALA-based PDD, HAL-based PDD, and NBI all resulted in a lower recurrence rate than WLC. **b** No difference in progression rate was observed between cancers resected by all methods investigated

**Fig. 8:**
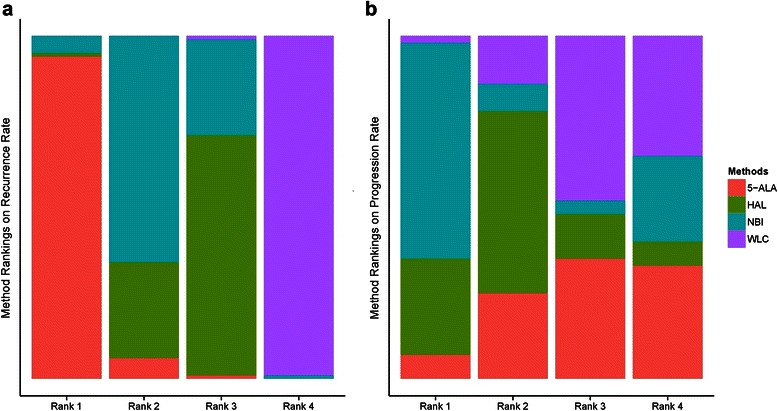
Rank probability test of network meta-analyses. **a** Cancers resected using 5-ALA-based PDD occupied the highest rank in the rank probability test followed by those resected using NBI. **b** NBI-assisted TUR was ranked highest in the rank probability test followed by TUR using HAL-based PDD

The recurrence rate of cancers resected using 5-ALA-based PDD was lower than that of those cancers resected using HAL-based PDD (*OR* = 0.48, 95 % CI [0.26–0.95]) and was not significantly different from those resected using NBI (*OR* = 0.53, 95 % CI [0.26–1.09]). The recurrence rates of cancers resected using HAL-based PDD versus NBI were also not significantly different (*OR* = 1.11, 95 % CI [0.55–2.1]). The use of 5-ALA-based PDD, HAL-based PDD, and NBI all resulted in a lower recurrence rate than WLC (Fig. 7a). Cancers resected using 5-ALA-based PDD occupied the highest rank in the rank probability test for recurrence rate, followed by those resected using NBI (Fig. 8a). No difference in progression rate was observed between cancers resected using 5-ALA-based PDD, HAL-based PDD, or NBI. Notably, TUR assisted by any of these techniques did not significantly decrease the rate of progression over WLC-assisted TUR ( Fig. 7b). NBI-assisted TUR was ranked highest in the rank probability test for progression-free rate, followed by TUR using HAL-based PDD (Fig. 8b); these rankings differed from those for recurrence rate.

## Discussion

Patients with tumors associated with CIS have a significantly greater risk of progression [23]. WLC is the current standard method for initial bladder cancer diagnosis but it has some disadvantages. These disadvantages can influence the planning and execution of TUR and may even influence the patient’s oncological outcomes. Bladder cancer diagnosis using a new video technology has recently being suggested as an alternative to overcome WLC’s disadvantages. Substantial research has been performed regarding diagnosis using a combination of PDD and NBI with the new video methodology.

PDD requires the instillation of a protoporphyrin derivative, typically a derivative of protoporphyrin IX (PpIX), and its selective uptake by dysplastic cells [24]. Under blue light, abnormal cells containing PpIX fluoresce red. The two most common agents used for PPD are 5-ALA and HAL, prodrugs that exhibit no photoactivity until they are metabolized in the cell. After uptake into the urothelial cell, they are incorporated in the conventional cellular hemobiosynthesis metabolism. The benefit of PDD to detect more bladder tumors and reduce residual tumors has been proven by previous meta-analyses [25–27]. The current study compares TUR assisted by PPD using 5-ALA or HAL to TUR assisted by other techniques, but the PpIX precursors’ efficacies were not compared to each other because no RCT has compared 5-ALA-based PDD and HAL-based PDD directly.

NBI, another optical enhancement technology, increases the contrast between vasculature and superficial tissue structures of the mucosa by excluding the red spectrum of white light. Early reports suggested that NBI improves detection of bladder tumors including CIS [24]. Two previous meta-analyses of NBI diagnostic accuracy demonstrated that NBI-assisted cystoscopy detects more NMIBC patients and tumors than WLC and that NBI effectively identifies abnormal lesions including CIS [28, 29]. While NBI-guided TUR has been reported to increase imaging quality versus unguided TUR, neither a meta-analysis nor a RCT comparing the two techniques has been performed.

A previous conventional pairwise meta-analysis of the therapeutic outcome in 12 RCTs by Yuan et al. demonstrated a low OR in the recurrence rate (*OR* = 0.5, 95 % CI [0.40–0.62]) after PDD-guided TUR. PDD-guided TUR also exhibited a low hazard ratio (HR) for recurrence-free survival (*HR* = 0.69, 95 % CI [0.53–0.77]). However, the authors cautioned that their meta-analysis did not distinguish TUR using 5-ALA-based PDD from that using HAL-based PDD and that the heterogeneity could affect the outcome [27]. Our study involved a network meta-analysis to overcome the heterogeneity described by Yuan et al. and was able to also analyze the outcome after NBI-guided TUR.

Although the recurrence rate of cancers resected using PDD or NBI has been shown in previous pairwise meta-analyses to be lower than that resected using WLC, a superior outcome in terms of progression rate has not been shown because less data concerning the rate of progression is available than data concerning the rate of recurrence; follow-up periods were relatively short in the enrolled studies. Second, because patients with high-risk tumors undergo adjuvant immunotherapy such as BCG instillation, differences in the rate of progression may be masked. Third, PDD and NBI exhibit high sensitivity and specificity toward the detection of CIS [30, 31], with an high area under curve of 0.939 for NBI [28], but these techniques may increase the rate of unnecessary biopsies.

Our analysis shows that resection using NBI and PDD did not differ significantly in terms of cancer recurrence rate. However, PDD-assisted TUR using 5-ALA was shown superior to that using HAL. In pair-wise meta-analyses, 5-ALA versus WLC showed an OR of 0.34 and 95 % CI of 0.22–0.51; meanwhile, HAL versus WLC showed an OR of 0.58 95 % and a CI of 0.45–0.74. In regards to ORs, that for 5-ALA was lower than that for HAL in conventional meta-analysis. In network meta-analysis, the OR was 0.48, which was similar to that in conventional meta-analysis; however, the 95 % CI was longer than that in conventional meta-analysis. The longer 95 % CI for the network meta-analysis was calculated by indirect comparison based on Bayesian networking.

A meta-analysis compared photosensitizing agents (5-ALA in 18 reports, HAL in five reports, and both in two reports) found similar sensitivity and specificity rates in patients (5-ALA versus HAL: sensitivity = 96 % versus 90 % and specificity = 56 % versus 80 %, respectively) and biopsies (5-ALA vs. HAL: sensitivity = 95 % versus 85 % and specificity = 57 % versus 80 %, respectively) [32]. Theoretically, compared to 5-ALA, HAL penetrates tissue more deeply and exhibits better accumulation in neoplastic cells [33]. However, the agent 5-ALA was initially developed for PDD and has therefore been evaluated in more studies than HAL [34]. The data reviewed seems to indicate some difference between the use of 5-ALA and HAL. Although the outcomes after TUR assisted by NBI or PDD do not seem to differ from each other, NBI-guided TUR is preferable to PDD because the specificity of PDD significantly decreases in patients who have undergone a recent instillation [35]. Accordingly, patients whose cancers are suspected to have recurred after intravesical therapy were helpful in evaluating the true specificity of NBI. Whereas PDD requires instillation of photosensitizing agents via a urethral catheter, NBI cystoscopy does not require extra invasive steps [36]. Recently, flexible cystoscopy was widely used to detect bladder tumors and to monitor bladder cancer patients; flexible cystoscopy is convenient in an outpatient setting.

## Conclusions

Previous RCTs and meta-analyses including the current study have proven PDD and NBI can enhance the diagnosis of bladder lesions, guide an adequate resection, and reduce tumor recurrence. In our network meta-analysis, TUR assisted by 5-ALA-based PDD demonstrated a lower recurrence rate than resection employing HAL. However, recurrence after resection using either 5-ALA or HAL was not significantly different than that after NBI-guided TUR. All new imaging technologies for bladder cancer were superior to WLC in lowering the recurrence rate, but did not improve outcome in terms of the progression rate.

## Abbreviations

NMIBC: Non-muscle invasive bladder cancer
TUR: Transurethral resection
WLC: White light cystoscopy
CIS: Carcinoma in situ
PDD: Photodynamic diagnosis
NBI: Narrow band imaging
5-ALA: 5-aminolaevulinic acid
HAL: Hexylaminolevulinate
RCT: Randomized controlled trial
PpIX: Protoporphyrin IX
